# Mortality rate and predictors among patients with breast cancer at a referral hospital in northwest Ethiopia: A retrospective follow-up study

**DOI:** 10.1371/journal.pone.0279656

**Published:** 2023-01-26

**Authors:** Mekides Misganaw, Haymanote Zeleke, Henok Mulugeta, Birtukan Assefa

**Affiliations:** 1 Department of Adult Health Nursing, College of Medicine and Health Science, Bahir Dar University, Bahir Dar, Ethiopia; 2 Department of Nursing, College of Health Science, Debre Markos University, Debre Markos, Ethiopia; 3 School of Nursing and Midwifery, Faculty of Health, University of Technology Sydney (UTS), Sydney, NSW, Australia; 4 Department of Pediatric Nursing, College of Health Science, Debre Markos University, Debre Markos, Ethiopia; Fondazione IRCCS Istituto Nazionale dei Tumori, ITALY

## Abstract

**Background:**

Breast cancer is one of the common global health concerns that affects2.1 million women each year and causes the highest number of cancer-related morbidity and mortality among women. The objective of this study was to determine the mortality rate and its predictors among breast cancer patients at the referral hospitals, in northwest Ethiopia.

**Methods:**

A retrospective follow-up study was conducted on breast cancer patients registered between February 01, 2015 and February 28, 2018. They were selected by simple random sampling using computer-generated method and followed until February 29, 2020, in Amhara region referral hospital. A pre-tested data extraction checklist was used to collect data from the registration book and patient medical records. The collected data were entered into Epi-Data version 3.1 and exported to STATA version 14 for analysis. The mortality rate by person-year observation was computed. The Kaplan-Meier survival curve with the log-rank test was used to estimate the survival probabilities of the patients. Bivariate and multivariate Cox regression model was used to identify predictors of mortality.

**Results:**

The overall mortality rate of breast cancer was 16.9 per 100 person-years observation. The median survival time was 38.3 (IQR: 26.23, 49.4) months. Independent predictors of breast cancer mortality was; Clinical stage IV and stage III (aHR:10.44,95% CI: 8.02,11.93 and aHR: 9.43, 95% CI: 6.29,11.03respectively), number of positive lymph node in the category of 10 and more and number of positive lymph node within the category of 4–9 (aHR:12.58, 95%CI: 5.2, 30.46 and aHR: 4.78, 95% CI: 2.19, 10.43respectively), co-morbidities (aHR:1.5, 95%CI: 1.01,2.21), Postmenopausal (aHR:2.03,95% CI: 1.37, 3), histologic grade III (aHR:2.12, 95% CI: 1.26,3.55) and not received hormonal therapy (aHR: 2.19, 95%CI: 1.52,3.15) were independent predictors of mortality.

**Conclusion:**

The overall mortality rate was 16.9 per 100 person-years. The finding was higher compared to high-income countries. Advanced clinical stage, co-morbidities, menopausal status, and hormonal therapy are the significant predictors of mortality. Early detection and treatment of breast cancer is needed to reduce the mortality rate.

## Background

Breast cancer is the leading cause of cancer-related death among women [1]. Globally, approximately 25% of cancer cases and 15% of cancer deaths among women were due to breast cancer. The mortality and incidence rate of breast cancer is highest in Africa, especially in the sub-Saharan African countries(SSA) [2]. According to the 2018 report by the World Health Organization (WHO), breast cancer is responsible for the deaths of 627,000 women worldwide. An ecological study showed that the highest standardized mortality rate in Eastern Europe was 18.6 per 100,000 and the lowest rate in Western Europe was 7 per 100,000 [3]. In Africa, the mortality rate was 17 per 100,000 and in eastern Asia, the mortality rate was 6.9 per 100,000 [4].

The five year survival status of patient with breast cancer was 84% in the US, 89.5% in Australia, 81% in Europe [5], 69.55 in Iran [6], 74% in Vietnam [7], 51.07% in Indonesia [8], 49.45 in Malaysia [9] and 66.1% in India [10]. Furthermore, a study conducted in Central Iran, shows that survival rates at one, two, three, four and five years were estimated at 98.0%, 96.0%, 92.0%, 89.0% and 87.0%, respectively [11]. Concerning Sub-Saharan countries the five year survival status was South Africa (53.4%), Gambia (11.9%), and Mali (13.6%) [19]. In Ethiopia A retrospective cohort study showed that the overall survival of breast cancer patients at the end of five years was 25.8% [12].

The survival of breast cancer patients was affected by many factors such as socio-demographic variables (age, educational level, financial status, family history), tumor pathological, and clinical parameters(tumor size, nodal status, presence of metastatic disease, clinical stage, tumor location, histology grade), the presence of co-morbidities and type of treatment [13–15].

Breast cancer is becoming a challenging health condition in Ethiopia with a high rate of morbidity and mortality [16]. The national crude death rate of breast cancer was 9.8 per 100 person- years [17]. It is also the most common type of cancer, accounting for more than one third of all cancer cases among women and one-fifth of all national cancer cases in the general population [18]. The average survival probability of metastatic breast cancer was about 12 months [19]. The overall median survival time was 56.5 months according to recent study conducted in a Black Lion specialized referral hospital [17].

Several strategies have been implemented to reduce mortality of breast cancer. Awareness creation strategy and implementation of breast cancer screening programs have been resulting in the reduction of mortality among women in Europe, North America, and Australia [20, 21]. However, the mortality rate in low and middle-income countries is high due to the lack of early detection and adequate treatment [22]. In Ethiopia, The national cancer control plan has the strategy to reduce the burden of breast cancer by promoting breast self-awareness and clinical breast examination for all women over the age of 18 [23].

Although breast cancer is the most frequent type of cancer in women and has high mortality rate. Limited evidence is available regarding the mortality rate and its predictors among Ethiopian breast cancer patients. Therefore, the objective of this study is to assess the mortality rate and its predictors among women with breast cancer at referral hospital in northwest Ethiopia.

## Methods

### Study design and population

**Fig 1:**
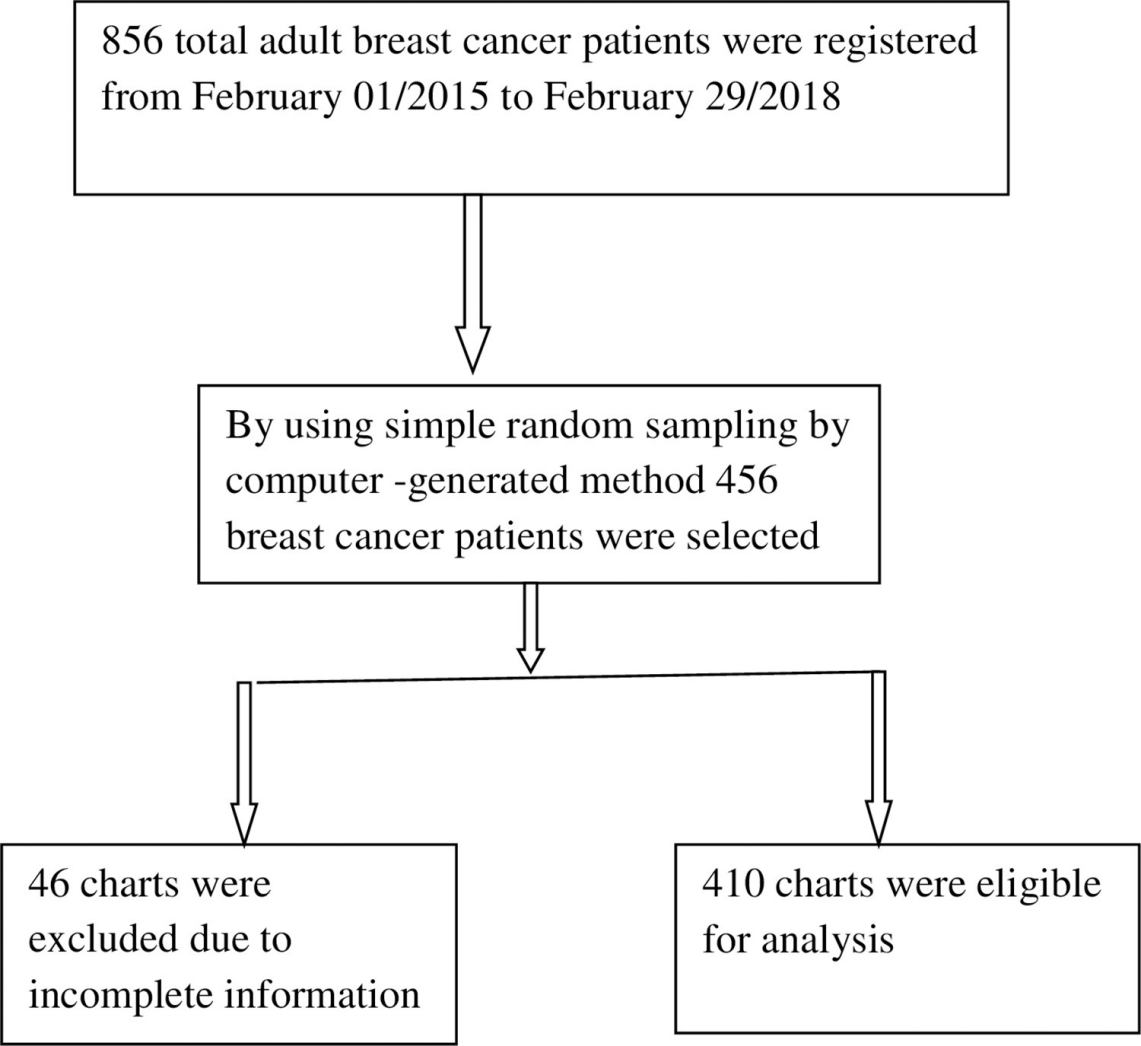
A flowchart that illustrates the inclusions and exclusions criteria.

A retrospective five-year institutional-based follow-up study was conducted among breast cancer patients in the oncology units of University of Gondar Comprehensive Specialized Referral Hospital (UOGCSRH). The University of Gondar Comprehensive Specialized Referral Hospital is located in Gondar town, 727.22 km from Addis Ababa, the capital city of Ethiopia. Gondar University comprehensive referral hospitals began cancer treatment in January 2015. All breast cancer patients who attended the oncology unit of UOGCSRH from February 01/2015 to feburary28/2020 were 856. To get a representative sample for the source population, the optimal sample size was calculated using the sample size determination formula for survival analysis with assumptions of 95% level of confidence, 80% power and 10% anticipated withdrawal follow-up was computed using the STATA Statistical package, Version14. The final sample size was 456. Simple random sampling by computer- generated method was used. The medical records of 410 patients were eligible for analysis [Fig 1].

#### Source population

All adult female patients diagnosed and enrolled in breast cancer treatment at the oncology units of UOGCSRH.

#### Study population

Adult female patients were selected by a simple random sampling method who were diagnosed and enrolled in breast cancer treatment at the oncology unit of UOGCSRH between February 01/2015 and February 29/2018 were included.

### Eligible criteria

#### Inclusion criteria

All adult female breast cancer patients who were newly diagnosed and enrolled in UOGCSRH during the study period (February 01/2015 to February 29/2018).

#### Exclusion criteria

✓ Whose medical charts were not found
✓ Incomplete charts

#### Operational definitions

**Adult:** a person who is over the age of 18.

**Censored:** Breast cancer women, who were transfer out, lost follow-up, died from another cause, and did not develop the event at the end of the follow-up period.

**Co-morbidities:** Any diseases found on the Charlson co-morbidities index. The presence of any of these conditions at diagnosis was denoted as ‘‘Yes” while the absence of these conditions at diagnosis will be denoted as ‘‘No”

**Event:** The occurrence of death at any time during the follow-up period after the confirmed diagnosis of breast cancer.

**Time to Event:** The time (in months) from the confirmed diagnosis of breast cancer to the date of death.

**Mortality rate:** The proportion of breast cancer patients who died during follow-up Period.

**Incomplete card:** when one of the basic independent variables like date of diagnosis, place of residence, stage at diagnosis, outcome of patient was not registered.

**Survival status:** The outcome of patients at the end of follow-up time could be dichotomized into censored or death that is sourced from the patient clinical data files from scheduled and unscheduled return visits.

### Data collection and statistical analysis

Trained Nurses extracted data from the patient’s chart using a standardized data extraction tool. Data about the time of breast cancer diagnosis, sociodemographic, clinical, and treatment-related factors of the patient were extracted. The data was coded, cleaned, and entered into EPI-data 3.1 and exported to STATA version 14 for analysis. Descriptive statistics were carried out to describe demographics, clinical and follow-up data in terms of central tendency and dispersion value for continuous data and frequency distribution for categorical data. Person-year of follow up was calculated using the time interval between the date of breast cancer diagnosis and the date of death or censor. A Kaplan-Meier (KM) survival curve was used to estimate the survival probability of death, and log-rank tests were used to compare the probabilities of survival curves between different categorical independent variables. The mortality rate and the cumulative survival probabilities were calculated. The assumption of a Cox regression proportional hazard model was cheeked using Schoenfeld residuals and Log-Log survival plot test. Overall model fitness was cheeked by the Cox-Snell residual with Nelson Aalen cumulative hazard. In bivariate analysis, independent predictors with a value of P-valueless than 0.25 were eligible for multivariable analysis. Adjusted hazard ratio (aHR) and its corresponding 95% confidence interval were used to declare a significant association between the outcome and independent variables. In multivariable analysis, variables with P-value <0.05 were considered statistically significant. The result of the study was presented with text, graph, and table.

## Results

### Sociodemographic characteristics of the study participants

A total of 410 adult patients were followed for a median follow-up time of 24.9 months with a minimum of 3.6 and a maximum of 60 months follow-up time after breast cancer diagnosis with a total observation time of 821 patient-year observation. The mean age of the participants at the time of diagnosis was 45 years with SD ± 12.22 years. A large proportion (n = 161, 39.27%) of the participants were in the age category of 41–55 years. More than half (n = 214, 52.2%) of the women were premenopausal (age less than 50 years old); slightly more than half (n = 234, 57.01%) of the participants were from rural areas. Furthermore, half (n = 213, 51.95%) of the women have a preexisting medical problem during the diagnosis of breast cancer on current with their breast cancer diagnosis (Table 1).

**Table 1:** Sociodemographic characteristics of the study participants.

Characteristics	Vital status at last contact		Total (%)
	Censored	Death (event)	
Age			
18–40	106(25.85%)	46 (11.2%)	139 (33.9%)
41–55	94 (22.93%)	67 (16.3%)	161(39.23%)
56–70	58(14.15%	24 (5.9%)	86(21.23%)
71–85	9(2.22%)	6 (1.46%)	11(2.75%)
Place of residence			
Rural	133(32.45%)	101(24.63%)	234(57.07%)
Urban	138(33.66%)	38(9.27%)	176 (24.93%)
Menopause status			
Premenopausal	169 (41.22%)	45(10.98%)	214 (52.2%)
Postmenopausal	102(24.88%)	94(22.93%)	196(47.81%)
Preexisting medical diagnosis			
Yes	99 (24.15%)	114 (27.8%)	213 (51.95%)
No	172 (41.95%)	25(6.1%)	197 (48.05%)

### Clinical, histopathological, and treatment characteristics

Approximately143 (44.88%) of the women were stage IV breast cancer at the time of diagnosis. Invasive ductal carcinoma (IDC) was the predominant histological type of cancer, 343 (83.66%). Almost one-third (31.46%) of the histology grade were moderately differentiated. Surgery with chemotherapy was the common mode of primary treatment initiated for patients following a diagnosis of breast cancer, affecting 254 (61.95%) patients. More than half, 212(64.83%) of the patients had modified radical mastectomy surgery. Almost half, 193 (47.07%) of the participants had received hormone therapy. More than half, 231 (56.34%) of tumor size was greater than 5cm during diagnosis (Table 2).

**Table 2:** Clinical, histopathological, and treatment characteristics of the participants.

Characteristics	Vital status at last contact		Total (%)
	Censored	Death	
stage of cancer at diagnosis			
I	10 (2.43%)	5(1.22%)	15 (3.66%)
II	93(22.68%)	5 (1.22%)	98 (25.85%)
III	127 (30.98%)	29 (7.07%)	156 (38.05%)
IV	35 (18.54%)	108(26.34%)	143(44.88%)
Histology type			
Invasive ductal	230 (67.05%)	113 (32.31%)	343 (83.66%)
Invasive Lobular	24(61.53%)	15(38.46%)	39(9.51%)
Invasive Medullar	20(71.42%)	10 (35.71%)	28 (6.83%
Deep surgical margin			
Free	199 (48.54%)	67 (16.34%)	266 (64.88%)
Involved	58 (14.14%)	86 (20.98%)	144(35.12%)
Node status			
Positive	60 (14.63%)	115 (28.05%)	175 (42.68%)
Negative	197 (48.05%)	38 (9.27%)	235 (57.32%)
Tumor size			
<2 cm	11 (2.68%)	6(1.46%)	17 (4.14%)
2–5 cm	120 (29.27%)	42 (10.24%)	162 (39.1%)
>5 cm	139 (12.68%)	97 (23.66%)	231 (56.3%)
Number of positive node			
1–3 nodes	210 (51.22%)	30 (7.32%)	240(58.54%)
4–9 nodes	35 (18.54%)	62 (15.12%)	97 (23.66%)
> = 10	26 (6.34%)	47 (11.46%)	73 (17.8%)
Type of surgery			
Partial mastectomy	29 (10.09%)	14 (4.28%)	43 (13.15%)
total mastectomy	33 (10.09%)	10 (3.06%)	43 (13.15%)
Modified radical mastectomy	148 (45.26%)	64 (19.57%)	212(64.83%)
Axillary node dissection	4 (1.22%)	8 (2.45%)	12 (3.67%)
Other	16 (4.89%)	1 (0.31%)	17 (5.2%)
Type treatment			
Chemotherapy	30 (7.32%)	33 (8.05%)	63 (15.37%)
Surgery with chemotherapy	161 (39.27%)	83 (20.24%)	244(59.5%)
Radiotherapy with Chemotherapy	9 (2.19%)	5 (1.22%)	14 (3.41%)
Surgery with chemotherapy and radiotherapy	43 (10.49%)	28 (6.83%)	71 (17.32%)
Palliative	7(1.7%)	9 (2.12%)	16(3.9%)
laterality of tumor			
Left	110 (26.83%)	87 (21.22%)	197(48.05%)
Right	131 (31.95%)	54 (13.17%)	185 (45.12%)
Bilateral	12 (2.93%)	12 (2.93%)	28 (6.83%)
Hormonal therapy			
Yes	163(48.37%)	30(8.9%)	193 (57.27%)
No	108(32.04)	109(32.34%)	217(64.38%)

### Mortality rate and survival status of patients with breast cancer

**Fig 2:**
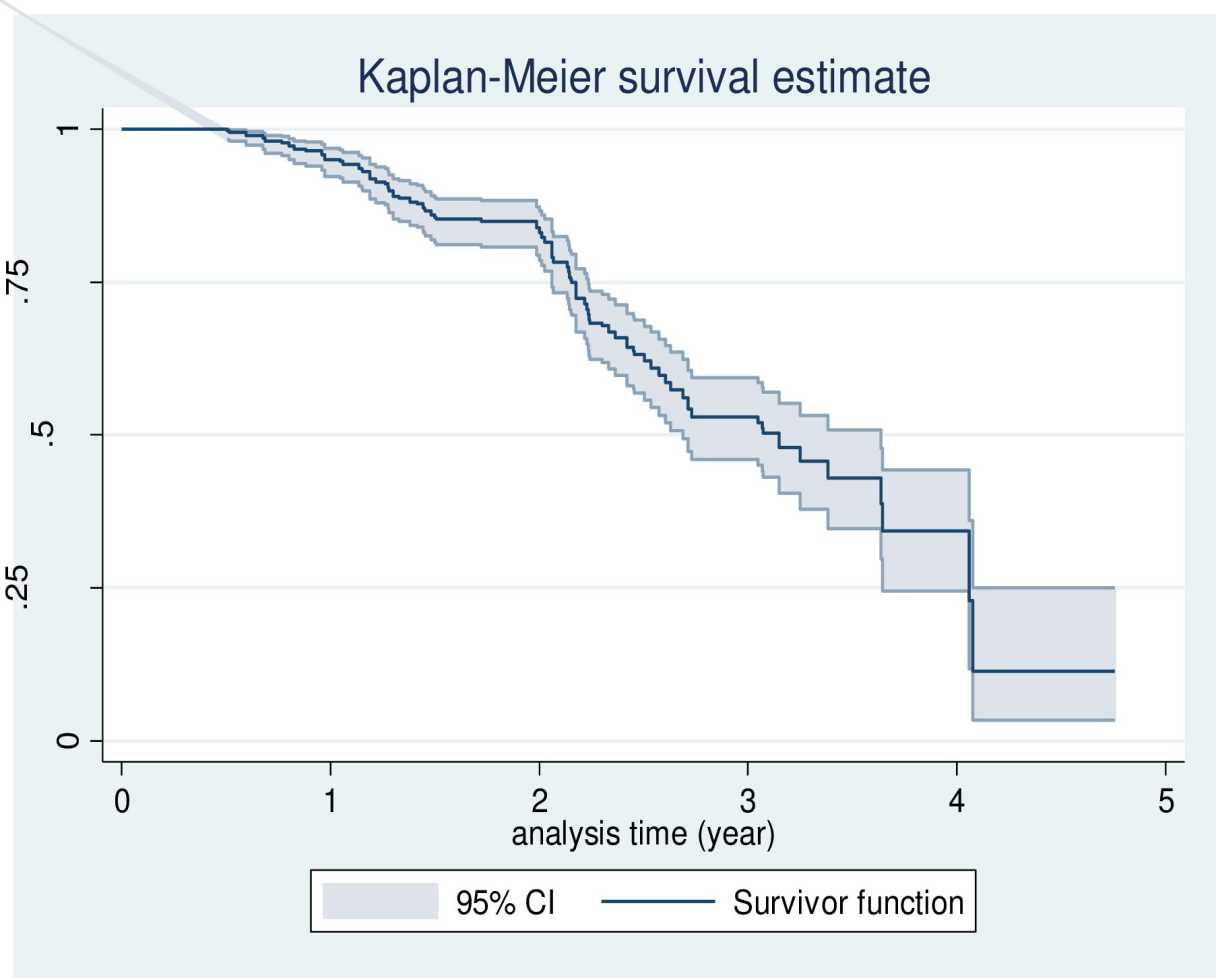
Kaplan-Meier estimation of survival functions among a cohort of breast cancer patients at the referral hospital in northwest Ethiopia.

A total of 410 participants were followed for a total of 60 months. The overall mortality rate was 16.9 (95% CI: 14.3, 19.9) per 100 person-year. The median survival time was 38.3(IQR: 26.23, 49.4) months. Kaplan-Meier survival estimation showed that the overall estimated survival probability after breast cancer diagnosis was 11.42% (95%CI: 9.37–24.94) at 60 months of follow- up. The Kaplan–Meier (KM) survival probability was 95% at 1 year, 83% at 2 years, 53% at 3 years, and 34% at 4 years after the diagnosis of breast cancer (Fig 2).

### Survival function among different groups of Breast cancer patients

Log-rank test was performed to test the equality of survival curves among various predictors for the presence of any significant differences in survival time (Table 3). The study showed that women with stage IV at baseline had a shorter median survival time (27.1 months, P-value: < 0.001, CI 95%:26.03,28.76) than those in early clinical stages (I, II) and stage II. Patient with co-morbidities had a shorter median survival time (28 months, P-value: < 0.001, 95% CI: 26.16, 31.7) than without co-morbidities. Furthermore, patients with histological grade I showed significantly better survival (49.6 month, P–value: <0.001, 95% CI: 47.2, 51.3) compared to those with other histologic grades. Women who had taken hormonal therapy had longer median survival time (46.86 months, P-value: <0.001, 95% CI: 44.26, 50.23). likewise, premenopausal women had better survival time (49.6 month, 95% CI; 33.2, 51.1) than postmenopausal women (31.3 month, p-value: = 0.001,95% CI; 27.2–38.3).

**Table 3:** Median survival time and log- rank test for the study population based on different characteristics of patients during 5-year of follow-up of breast cancer patients at, UOGCSRH.

Characteristics	Survival time(month), (95% CI)	Overall 5 year	Log-rank test
	Median (95% CI)	Survival(%)	(p-value)
Residence			
Urban	45.2(32.41,48.64)	59.11 (44.94,70.78)	18.48(0.001)
Rural	30.8 (28.76,33)	11.20 (3.53, 23.86)	
Menopause status			
Premenopausal	49.6 (33.2,51.1)	41.92(17.79, 64.55)	16.52(0.001)
Postmenopausal	31.3 (27.2,38.3)	8.64 (1.88, 22.13)	
Co-morbidity			
No	49.4(44.26,50)	61.19(39.73,76.99)	61.63(0.001)
Yes	28(26.16,31.7)	7.8(2.32, 10.94)	
Stage			
I		100	118.52(0.001)
II	55.2(50,57.4)	96.87 (87.6, 99.24)	
III	44.3(41.16,46.3)	34 (26.18,46.89)	
IV	27.1(26.03,28.76)	8.32(2.91, 11.48)	`
histology grade			
Grade I	49.6(47.2,51.3)	53.21(35.43,68.14)	18.05(0.001)
Grade II	49.4(45.83,50.68)	35(16.97, 53.72)	
Grade III	37.36(32.43,39.78)	16.84(9.12, 27.04)	
Deep surgical margin			
Free	42.2(39.53,43.56)	55.33(43.71,65.49)	36.38(0.001)
Involved	28.36(26.1,31.3)	30.09(20.46,40.28)	
Number of involved			
lymph nodes			
< = 3	35.2(31.25,39.65)	67.7(50.93, 79.82)	70.37(0.001)
4–9 no	29.43(26.46,32)	25.7(15.93, 36.74)	
> = 10	27.1(25.06,30.46)	23.43(12.68,36.08)	
Tumor size			
<2			
2–5	49.4(42.3,53.2)	37.1(27.66, 46.54)	20.09(0.001)
> = 6	31.7(29.43,32.33)	28.17(18.75, 41.27)	
Endocrine therapy			52.2(0.001)
Yes	46.86(44.26,50.23)	60.05(41.5, 74.4)	
No	29.43(27.1,31.7)	8.07(2.39, 10.35)	
Lymph node status			
Negative	49.4(39.62,53.64)	75(31.48, 93.09)	5.93(0.041)
Positive	37.63(32.7,41.16)	24.94(13.17, 28.64)	

CI; confidence interval

### Predictors of mortality

Advanced clinical stage, high histologic grade, place of residence, co-morbidities, menopausal status, number of positive lymph nodes, and hormonal therapy were significant predictors of mortality among breast cancer patients (Table 4).

**Table 4:** Bivariable and multivariable Cox regression analysis for predictors of mortality among breast t cancer patients in UOGCSRH from February 2015 to February 2020.

Characteristics	Survival status		Bivariable	Multivariable
	Death	Censor	cHR (95% CI	aHR (95% CI)
Place of residence				
Urban	38(9.27%)	138(33.66%)	1	1
Rural	101(24.63%)	133(32.45%)	1.74(1.26,2.4)*	1.25(0.88,1.78)
Menopause				
Premenopausal	45(10.98%)	169(41.22%)	1	
Postmenopausal	94(22.93%)	102(24.88%)	2.46 (1.65,3.98)*	2.03(1.37,3)**
Comorbidit				
No	25(6.1%)	172(41.95%)	1	
Yes	114(27.8%)	99(24.15%)	2.304 (1.61,3.3)*	1.5(1.01,2.21)**
Stage of breast cancer				
I	5(1.22%)	10(2.43%)	1	1
II	5 (1.22%)	93(22.68%)	2.93(0.02,5.64)	2.54(0.62,6.02)
III	29 (7.07%)	127(30.98%)	9.88 (6.37,12.79)*	9.43(6.3,11.03)**
IV	108(26.34%)	35(18.54%)	11.01(4.73,11.17)*	10.44(8.03,11.03)**
Histology grade				
Grade I	9(20.1%)	34(79.1%)	1	1
Grade II	29(25%)	87(75%)	1.86(0.4,2.22)	1.32(0.77,2.22)
Grade III	71(28.28)	150(59.76)	2.88(1.25,11.23)*	2.12(1.26, 3.55)**
No of involved lymph node				
< = 3	30 (7.32%)	210 (51.2%)	1	1
4–9 nodes	62 (15.12%)	35 (18.54%)	5.27(3.87,9.68)*	4.78(2.19, 10.43)**
> = 10	47 (11.46%)	26 (6.34%)	13.6(7.27, 20.8)*	12.58(5.19,30.46)**
Lymph node status				
Negative	115(28.05%)	60 (14.63%)	1	1
Positive	38 (9.27%)	197(48.05%)	1.92(1.13,2.41)*	1.68(0.56,5.05)
Hormone therapy				
Yes	30(15.54)	163(84.4%)	1	1
No	109(50.23) %)	108(49.76%)	2.87(2.05, 4.02)*	2.19(1.52, 3.16)**

CI; confidence interval, aHR; adjusted Hazard ratio, cHR: crude hazard ratio

* indicates that the variables significantly associated with the outcome at bivariable analysis 95% level of significant (< 0.05)

** indicates that the variables significantly associated with the outcome at multivariable analysis 95% level of significant (< 0.05).

Inthe multivariable Cox regression modelthe hazard ratio for stage IV was 10.44 times higher than that for stage I (aHR: 10.44, 95%CI: 8.03, 11.94). Likewise, the hazard ratio for stage III was 9.43 times higher than that for stage I (aHR: 9.43, 95% CI: 6.3, 11.03). Additionally, women who had not received endocrine therapy during the follow-up time were 2.19 times more likely to die as compared to those who had taken endocrine therapy (aHR: 2.19, 95%CI: 1.52, 3.15). Women who had one or more co-morbidities on the Charles co-morbidities index were 1.5 times more likely to die to compared to their counter parts (aHR: 1.5, 95%CI: 1.0, 2.21). The hazard ratio for patients with positive lymph node > = 10 was 12.58 times higher than that having < = 3 positive lymph node at the time of diagnosis. (aHR: 12.58, 95%CI: 5.19,30.46). Likewise, patients who had positive lymph nodes in categories 4–9 were more than 4.78 folds to die compared to those patients having < = 3 positive nodes (aHR: 4.78, 95% CI:2.19, 10.43). Women with a histologic grade III at the beginning of breast cancer diagnosis had the hazard of 2.12 times to die as compared to those who were grade I (aHR: 2.12:, 95% CI:1.26, 3.55). Furthermore, postmenopausal women were 2 times more likely to die compared to premenopausal women (aHR: 2.03, 95% CI: 1.37, 3)

### Testing of overall model fitness

**Fig 3:**
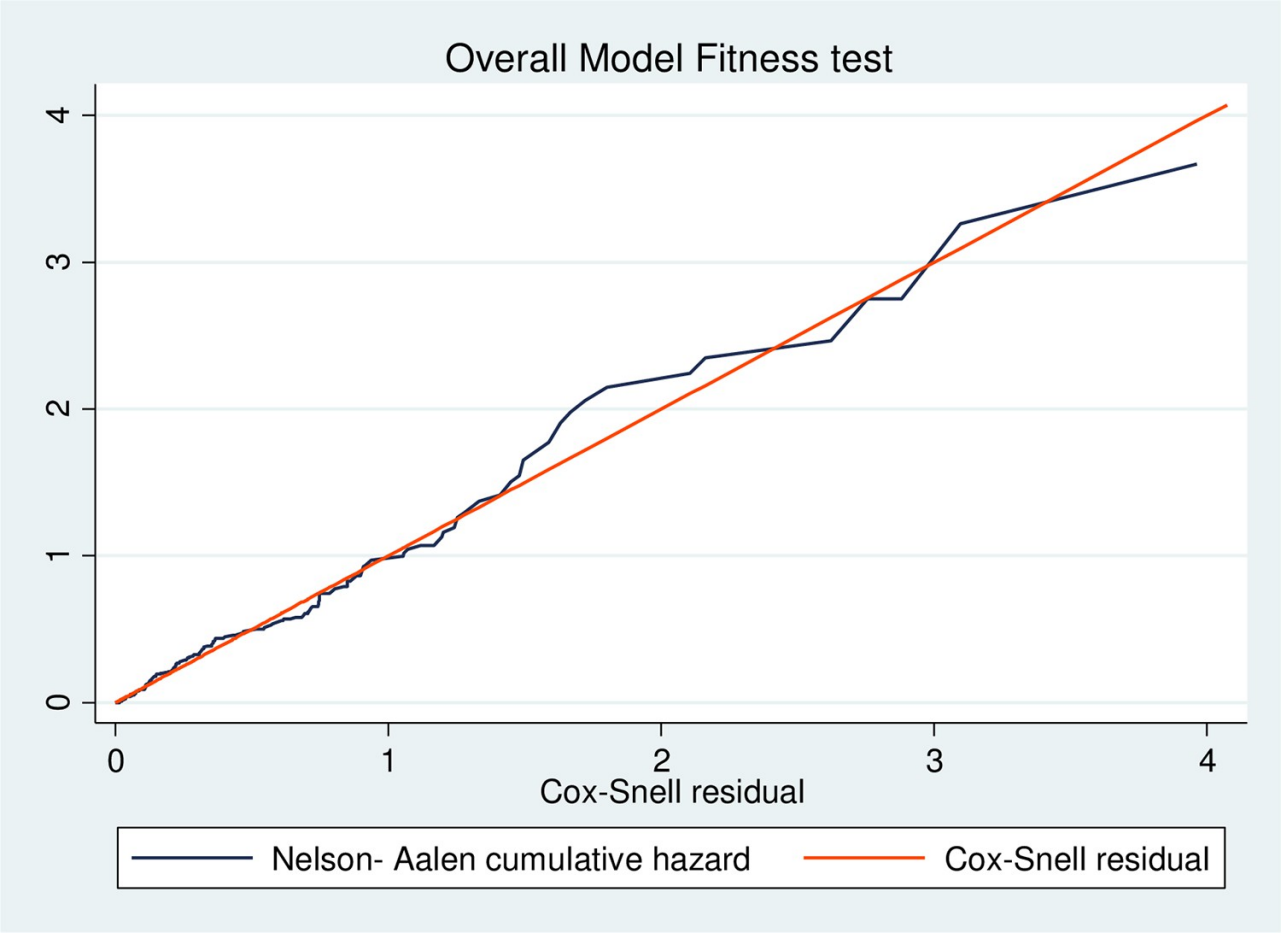
Model adequacy for incidence of mortality and its predictors of breast cancer patients at the referral hospital in northwest Ethiopia.

The Cox-Snell residual plot was used to test the fitness of the model. The figure shows that if the Cox regression model fits the data, these residuals should have a standard censored exponential distribution with a hazard ratio. The hazard function follows the 45° line closely. Therefore, the output shows that the Cox–Snell residuals were satisfied with the overall fitness test of the model (Fig 3).

## Discussion

This study aimed to assess the mortality rate and its predictors among breast cancer patients. The result showed that the overall mortality rate was 16.9per100 person-year (95% CI: 14.3, 19.9). This mortality rate was higher than study done in central Ethiopia, which reported 9.8 per 100 patient-year [17]. The higher mortality rate in our study could be due to co-morbidity conditions and advanced clinical stage of the study participant. Having one or more co-morbidities was linked to significantly increased the mortality rate for breast cancer survivors, which agrees with previous studies [2]. The finding of the current study is in line with the cumulative incidence of mortality in Nigeria 16.2 per 100 person-years [24] and in Cameron 11 per person-year. However the incidence of mortality was higher than in studies done in North Africa 29.3 per 100, 000 person years and 22.4 per 100, 000 person-years in SSA [25], western countries [5, 15, 26]. The higher incidence of mortality in our study could be due to the advanced stage of the disease at diagnosis [27]. Furthermore, the discrepancy in the results could be due to differences in the sample size, follow-up period, characteristics of the study participants, the prevalence of breast cancer and difference in treatment modality [14].

In the present study a median survival time was 38.3 (95% CI, 32.7, 44.2) months. The overall survival rate was 11.42% at 60 months of follow-up. This finding is consistent with a similar study conducted in Gambia, which was 11.9% and Mali which was 13.6% [28]. However, this finding is lower compared with other similar studies conducted in an Asian countries (56.04%) [29], Sweden (89%), Canada (86%), and United States (88%) [30]. The lower survival rate of breast cancer in the developing countries compared to some western and Asian countries could be due to the limited availability of adequate staging and treatment therapy, the absence of early screening and detection [5].

In this 5-year retrospective follow-up study, the overall survival rates at 1, 2, and 3 years were 95%, 83%, and 53%, respectively. This finding is consistent with other previous studies conducted in central Ethiopia (97.2%, 80.8%, and 46.2%) [17] Malaysia (70.8%, 56.9%, and 49.4%) [9] and in Vietnam (94%,83%,74%) [7]. However, this figure is higher than those high- income countries such as Northwest Iran (98%, 96%, and 92%), US (98%,86%,83%), and Germany (83.61%,67.53%, and58.75%) [5, 6, 11]. The difference could be due to the fact that the current study was conducted in a high-level specialized hospital that provides services for patients referred from general hospitals with advanced disease stages and histological grades.

In the current study, overall median survival decreased with advanced disease, (100% for stage I, and, only 8.32% for stage IV). This figure is similar to a study conducted in Sudan (100% for stage I, and only 5% for stage IV) [31], in Ethiopia (92.86%, and 16.8%) [17], in Gahanna (91.94%, and 15.09%) [32]. Early stages of the diseases with better survival were observed.

This could be indicating that early detection and treatment greatly improve the survival probability of women with breast cancer. This study did not show significant differences in survival between the different age categories which could be due to a high proportion of stage III and stage IV breast cancer in our study population which worsened the outcome [14, 33].

Regarding predictors of mortality, women who had not received endocrine therapy during the follow-up time were more than 2 folds to die compared to those women who had received endocrine therapy. This finding is in agreement with a similar study conducted in Ethiopia [17], and in other African countries [9, 13, 34]. The effect of hormone therapy on survival improvement was due to the influence of hormone receptors [35]. Risk of dying among postmenopausal women was twice as higher than among premenopausal women. This finding is consistent with different studies conducted in an Asian countries [5, 6, 30]. This might be due to most postmenopausal women are obese [36], leading to high estrogen production in adipose tissue with consequent increase in the available biological circulating estradol, which has promotional effect on breast carcinogenesis [37]. Furthermore, women who had histologic grade III at diagnosis died 2.11 times compared to women who had grade I. This finding was consistent with other previous studies which have been conducted in Ethiopia [17] and Asian countries [30, 38, 39]. This could be due to high-grade cancer cells tending to be more aggressive and have a bad prognosis [40]. This indicated that the assignment of histological grades has been consistent between pathologists. Similarly, women with stage IV were more than 10 times more likely to die compared to those with stage I. This finding was consistent with a similar study conducted in Ethiopia [17], Cameroon [24], Sudan [31]. Stage is an important predictor of survival from breast cancer. It should be noted that there is a considerable delay between the first symptoms and the presentation to health care practitioner. Some reasons for late presentation in African countries include low awareness of breast cancer, difficulty to access healthcare and pathways within the healthcare system often hinder early diagnosis [41–43]. Furthermore, our study indicates that co- morbidities in breast cancer patients were strongly related to the risk of death. This result was consistent with a similar study conducted in Ethiopia [17] and Western countries [44–46]. This might be due to patients with co-morbidities were more physiologically vulnerable to treatment toxicity from the current or previous cancers [47]. In addition, co-morbidities associated with deference in morphology, histology, differentiation, and proliferation status of cancer cell [48]. Furthermore, patients with an increase in the number of positive lymph nodes involved had a higher risk of death. This is due to an increased relapse rate that decreased survival rate [49].

## Conclusions

The findings of this study revealed that the overall incidence of mortality was higher compared to those of high- and middle-income countries. Advanced clinical stage, high histologic grade, place of residence, co-morbidities, menopausal status, number of positive lymph nodes, and hormonal therapy were significant predictors of mortality among breast cancer patients. Early detection and adequate treatment programs must be implemented to reduce the mortality rate.

## Supporting information

S1 File (DTA)

## Abbreviations

aHR: adjusted hazard ratio
GLOBOCAN: Global burden of cancer
HIC: high-income country
IDC: invasive ductal carcinoma
LMIC: low- and middle- income countries
SSA: Sub-Saharan Africa
USA: United States of America
UOGCSRH: University of Gondar compressive specialized referral hospital
WHO: world health organization
